# Serum cholinesterase as a biomarker for sepsis-associated immunosuppression via Hub gene RORA

**DOI:** 10.1371/journal.pone.0348979

**Published:** 2026-05-07

**Authors:** Qian He, Xu Huang

**Affiliations:** 1 Department of Emergency, The Second Affiliated Hospital of Chongqing Medical University, Chongqing, People’s Republic of China; 2 Department of Emergency, The First Affiliated Hospital of Chongqing Medical University, Chongqing, People’s Republic of China; China Academy of Chinese Medical Sciences Institute of Chinese Materia Medica, CHINA

## Abstract

**Background:**

Sepsis-induced immunosuppression is a key factor contributing to high mortality rates. However, suitable biomarkers for routine clinical monitoring of immune function are currently lacking. Serum cholinesterase levels are markedly diminished in sepsis and are associated with unfavorable prognoses, its role in the immunosuppression pathology and the mechanisms involved remain inadequately understood.

**Methods:**

We conducted a translational study integrating clinical research, bioinformatics analysis and animal experiments. Initially, within a single-center clinical cohort, we investigated the correlation between serum cholinesterase levels and lymphocyte subsets in patients suffering from sepsis, subsequently evaluating its association with disease severity (APACHE-II and SOFA scores) and clinical outcomes. Subsequently, by integrating sepsis transcriptome data with cholinergic anti-inflammatory pathways and immune-related gene sets, we identified the hub gene RORA and validated it across multiple dimensions using public databases. Finally, in the CLP sepsis mouse model, we measured cholinesterase activity and specifically quantified RORA mRNA expression in the spleen. We then analyzed the correlation between these measurements and changes in key immune cell counts.

**Results:**

Clinical data revealed significantly reduced serum cholinesterase activity in sepsis patients. Decreased cholinesterase levels positively correlated with elevated disease severity scores (APAChE-II, SOFA) and reduced counts of CD4 ⁺ T cells, CD8 ⁺ T cells, and NK cells. Bioinformatics analysis identified RORA as a hub gene linking sepsis, cholinesterase, and immune responses. Across eight independent GEO datasets, RORA expression exhibited a consistent downregulation trend in sepsis with high diagnostic value. Analysis of immune cell infiltration revealed significant positive correlations between RORA and counts of CD4 ⁺ T, CD8 ⁺ T, and NK cells in sepsis. In the CLP mouse model, reductions in spleen CD3 ⁺ T, CD4 ⁺ T, and CD8 ⁺ T cell counts coincided with notable decreases in serum cholinesterase and spleen RORA mRNA levels. Both serum cholinesterase concentration and spleen RORA mRNA expression exhibited positive correlations with CD4 ⁺ T and CD8 ⁺ T cell counts.

**Conclusion:**

This study establishes serum cholinesterase as a valuable clinical biomarker for assessing sepsis diagnosis, disease severity, and immunosuppression. For the first time, through multiomics integration and experimental validation, RORA has been identified as the key molecular bridge linking the cholinesterase activity and immunosuppression in sepsis. This not only provides a new direction for understanding immune dysregulation in sepsis but also lays a theoretical foundation for the future development of RORA-targeted immunomodulation and treatment strategies for sepsis.

## Introduction

Sepsis, characterized by life-threatening organ dysfunction due to a dysregulated host response to infection, remains one of the foremost causes of mortality in intensive care units globally. Despite advances in supportive care and antibiotic therapy, the persistent high mortality rate underscores our incomplete understanding of its complex pathophysiology [1–3]. Notably, beyond the initial hyperinflammatory phase, a prolonged state of immunosuppression is now recognized as a critical hallmark of sepsis, characterized by widespread lymphocyte apoptosis, functional exhaustion, and an increased susceptibility to secondary infections [4,5].

Accurately assessing the immune status of septic patients is paramount for implementing precise immunomodulatory therapies. However, the current clinical arsenal lacks ideal biomarkers for routine immune monitoring [6]. While flow cytometry can precisely quantify lymphocyte subsets, its complexity and cost hinder its use for dynamic assessment. Consequently, there is an urgent clinical need to identify easily measurable serum biomarkers that reliably reflect the degree of immunosuppression.

Cholinesterase (ChE) has traditionally been used as a marker to evaluate hepatic synthetic function and nutritional status. However, our findings show that serum ChE levels are often significantly reduced in patients with sepsis. Moreover, the extent of this reduction closely correlates with disease severity and mortality rates. Intriguingly, beyond its metabolic role, ChE is integral to the cholinergic anti-inflammatory pathway (CAP) by hydrolyzing acetylcholine, the key neurotransmitter that suppresses pro-inflammatory cytokine release from macrophages via α7 nicotinic acetylcholine receptors [7–9]. The significant decline in ChE activity, observed during sepsis, suggests that this reduction is not merely a secondary effect but may actively contribute to immune dysfunction by disrupting the regulatory balance of the CAP. While clinical observations have hinted at a correlation between low ChE levels and lymphopenia, the precise molecular mechanisms linking ChE activity to immune cell depletion remain entirely elusive [10]. Therefore, we hypothesized that the decrease in ChE disrupts cholinergic immunomodulation, ultimately leading to immunosuppression, and that this process is mediated by specific hub genes.

To test this hypothesis, we designed an integrated study. First, a clinical cohort study was conducted to corroborate the association between serum ChE levels and indicators of immunosuppression. Subsequently, we applied a bioinformatics strategy to analyze sepsis transcriptomic datasets, aiming to objectively identify hub genes that mediate interactions between the cholinergic system and immune response pathways. Finally, the functional role of the key hub genes were experimentally validated in a murine model of sepsis. This multi-faceted strategy aims to unveil a novel mechanism underlying sepsis-induced immunosuppression, potentially identifying new targets for diagnostic and therapeutic strategies.

## Materials and methods

### Ethics statement

The study was conducted in accordance with the Code of Ethics of the World Medical Association. The study protocol was reviewed and approved by the Ethics Committee of the First Affiliated Hospital of Chongqing Medical University (Approval No. 2025-862-01). Informed consent was waived by the Ethics Committee. Studies involving mice were conducted in strict accordance with the ethical guidelines of the Laboratory Animal Center of Wuhan Servicebio Technology Co., Ltd. The experimental protocols complied with the ARRIVE guidelines (Animal Research: Reporting of In Vivo Experiments) and were approved by the Institutional Animal Care and Use Committee (IACUC) of Wuhan Servicebio Technology Co., Ltd. (Ethics Reference Number: 2024192). All animal experimental procedures were performed in accordance with the Guide for the Care and Use of Laboratory Animals by the National Research Council.

### Study design and case selection

This was a single‑center retrospective cohort study. Only de‑identified data with all personal identifiers removed prior to analysis were used. Consecutive adult patients (aged ≥ 18 years) admitted to the Emergency Intensive Care Unit (EICU) of the First Affiliated Hospital of Chongqing Medical University between November 13, 2025 and December 31, 2025 were enrolled. Patients were divided into sepsis and control groups according to Sepsis‑3 criteria. The control group comprised critically ill patients without evidence of infection, including severe trauma, acute pancreatitis, gastrointestinal bleeding, and stroke. Exclusion criteria were: age < 18 years, chronic liver disease, cirrhosis, acute liver failure, organophosphorus poisoning, and missing key data.

### Data collection

Data were collected independently by two attending physicians specializing in emergency and critical care medicine, who were blinded to the study hypothesis. They collected the data by reviewing the electronic medical record system. Collected data encompassed baseline characteristics (age, sex) and disease severity scores. Specifically, the Acute Physiology and Chronic Health Evaluation II (APACHE II) score and the Sequential Organ Failure Assessment (SOFA) score were recorded within 24 hours of admission. All patients were diagnosed with sepsis within 24 hours of admission according to Sepsis-3 criteria, and all laboratory indicators were measured within 24 hours after diagnosis. Clinical outcome was all-cause in-hospital mortality, including routine biochemical parameters, inflammatory markers, absolute lymphocyte count in peripheral blood, and serum ChE activity. All blood samples were analyzed uniformly by the hospital’s Clinical Laboratory Department. Serum ChE activity was measured using the enzymatic rate method, with a normal reference range of 5000–12220 U/L.

### Microarray data

The RNA-sequencing datasets used in this study were downloaded from the Gene Expression Omnibus (GEO) database of the National Center for Biotechnology Information [11]. The GSE137340 dataset included array-based gene expression profiles of whole blood from 15 sepsis patients at the time of diagnosis and 12 healthy control subjects under the platform GPL10558. In addition, more sepsis datasets were also collected for the following independent external validation to verify the diagnostic performance of sepsis. The array-based gene expression profile dataset GSE69528 included whole

blood samples from 83 septic patients and 55 uninfected controls. The array-based gene expression profile dataset GSE13904 included whole blood samples from 52 septic children, 106 septic shock children and 18 normal children. The array-based gene expression profile dataset GSE25504 included whole blood samples from 25 sepsis human neonates and 37 controls. The array-based gene expression profile dataset GSE26440 included whole blood samples from 98 septic shock children and 32 controls. The array-based gene expression profile dataset GSE28750 included whole blood samples from 10 septic patients and 20 controls. The array-based gene expression profile dataset GSE145227 included 10 septic children and 12 controls. The array-based gene expression profile dataset GSE72326 included whole blood samples from 15 septic patients and 21 controls. The array-based gene expression profile dataset GSE95233 included whole blood samples from 51 septic patients and 22 controls.

### Screening for Differentially Expressed Genes (DEGs)

DEGs between sepsis patients and control individuals in GSE137340 were screened by the “limma” package and volcano plots of DEGs were plotted using the “ggplot2” package to show the differential expression.

### Screening of candidate hub DEGs

From the Immunology Database and Analysis Portal (IMMPORT) database (https://www.immport.org/home), we downloaded totaling 2483 immune-related genes (IRGs). 1140 Cholinergic anti-inflammatory pathway related genes (CAPRGs) were obtained from the GeneCard database(GeneCards – Human Genes | Gene Database | Gene Search). The overlapping genes between DEGS, IRGs and CAPRGs were identified as candidate hub DEGs by Venn diagram analysis, and were analysed by R language and cluster analysis packages for subsequent Gene Ontology (GO), Kyoto Gene and Genome Encyclopedia (KEGG) pathway enrichment analysis. The analysis of PPI, MCODE and the function enrichment of hub DEGs were obtained by The Metascape database (http://metascape.org/).

### Screening key genes through LASSO machine learning algorithms

The LASSO regression analysis was performed using the glmnet package (v 4.1−4), with the

parameter setting of family = binomial [12]. Then, 10-fold cross validation was applied, and the L1-penalty (lambda) was used to shrink less important genes to zero. The error rate was calculated for each lambda value, and the optimal lambda was identified. The genes whose regression coefficients were not penalized to zero were selected as the more important feature genes for the disease, and the best classification model was constructed. Furthermore, key genes were obtained by LASSO machine learning algorithms.

### Screening of hub DEG

Key genes were screened out using LASSO regression analysis. Hub gene RORA was then identified as the overlapping gene between candidate hub DEGs and key genes using Venn diagram analysis.

### Single-gene analysis for the RORA

On the basis of the median value of RORA expression, sepsis samples in the dataset GSE137340 were classified into the RORA high expression group and the RORA low expression group. Differentially expressed genes (DEGs) between these two groups were identified using the R package DESeq2, and volcano plots showing differential expression were generated using the ggplot2 package. These DEGs were also analyzed using R and clustering analysis packages for Gene Ontology (GO) and Kyoto Encyclopedia of Genes and Genomes (KEGG) enrichment analyses.

### Gene set enrichment analysis

To identify biological signaling pathways, GSEA was performed between high and low levels of RORA expression in sepsis samples from the GSE137340 dataset. The KEGG pathways showed significant enrichment based on net enrichment scores (NES), gene ratios, and P-values. Statistical analyses were conducted using R software, and the results were visualized with the ggplot2 package.

### Analysis of the immune microenvironment

An immune cell infiltration analysis was performed to assess the relative abundance of immune cells in sepsis and control samples, utilizing the CIBERSORT algorithm based on 22 immune cell gene markers. The distribution of immune cell components across each sample was visually depicted using bar plots, while differences in immune cell infiltration between the two groups were compared employing the Wilcoxon rank-sum test. Additionally, Spearman correlation analysis was performed to investigate the relationship between RORA expression and immune cell infiltration, with visualizations generated using the R (4.2.1) package “ggplot2.”

### Validation of RORA expression and diagnostic value

The mRNA expression of RORA was verified in GSE137340, GSE95233, GSE72306, GSE69528, GSE28750, GSE13904, GSE25504 and GSE145227. The comparison between the septic patients and controls was conducted with the T-test. Statistical significance was set at p-values less than 0.05. We determined the sensitivity and specificity of RORA by performing Receiver Operating Characteristic (ROC) curve analysis with the R pROC package, visualized using the R package “ggplot2”.

**scRNA-seq data analysis**

Single-cell RNA sequencing (scRNA-seq) data from the GEO database, specifically the septic patient dataset GSE175453, which includes five control samples and four disease group samples, were analyzed to explore the expression of key genes at the cellular level. Initially, scRNA-seq data were filtered using the Seurat package (v5.0) to exclude cells with fewer than 300 genes and genes represented in fewer than five cells [13]. The retained genes and cells met the following criteria: 200 < nFeature < 3,000, nCount < 20,000, and mitochondrial percentage < 5%. Subsequently, multiple samples were amalgamated utilizing the IntegrateData function, and the filtered dataset underwent normalization through the NormalizeData function from the Seurat package. Subsequently, the identification of 2,000 highly variable genes was performed using the FindVariableFeatures function. Following this step, principal component analysis (PCA) was conducted to evaluate the distribution of the 2,000 highly variable genes across the normal control (NC) and sepsis disease cohorts. The data normalization process was further refined using the ScaleData function within the Seurat package, and the statistically significant principal components (PCs) were identified utilizing the JackStrawPlot function. Subsequently, cell clustering was executed employing the Uniform Manifold Approximation and Projection (UMAP) method, with a resolution parameter set at 0.4. Furthermore, the resultant cell subpopulations were annotated through the application of the SingleR package (version 1.0.6) to ascertain specific cellular types [14].

### Cell communication analyses

The CellChat package (v1.6.1) [15] was employed for cell communication analysis in annotated cell types. Following the creation of CellChat objects, the importation of ligand-receptor data from CellChatDB.human, and preprocessing, cell communication networks were generated. Heat maps and circle plots were utilized to visually represent the number and weight of interactions among cell types, while bubble plots were constructed to demonstrate the probability of communication mediated by specific ligand-receptor pairs from particular cell populations to other cell groups. Subsequently, the expression of key genes in different cell types was demonstrated by UMAP, followed by implementing the Wilcoxon test to assess differences in key gene expression among cell types. Furthermore, the subcellular localization of RORA was predicted based on the COMPARTMENTS database (https://compartments.jensenlab.org/) to provide a basis for understanding the changes in RORA in the disease process.

### Animal

Eight-week-old male C57BL/6 mice were housed according to internationally accepted standards, with four animals per cage, under constant ambient temperatures of 22 ± 0.5 °C. A 12-h light/12-h dark photoperiod schedule was strictly maintained to preserve their circadian rhythms. Adequate food and water were provided adlibitum. All efforts were made to minimize suffering, including post-operative monitoring and appropriate analgesia. Mice were euthanized with an overdose of isoflurane.

### Constructive of sepsis mice model

According to the experimental design, C57BL/6 mice were randomly divided into two groups, including the sham group and the cecal ligation and puncture (CLP) surgery group. There were 10 mice in each group. As previously described [16], mice were anesthetized by inhalation of 3–4% isoflurane. Then, a mid-abdominal incision was made. Ibuprofen (250 μg/mL in drinking water) was used for pre- and post-operative analgesia. Exposure, ligation, and double puncture of the cecum were accomplished using a 21-gauge needle, followed by gentle extrusion of a small amount of fecal content. Subsequently, the cecum was repositioned and sutured back into the abdomen. Mice in the sham group underwent identical surgical techniques, except that the cecum was exposed but neither ligated nor punctured. Immediately following surgery, all mice received a subcutaneous injection of 0.5 mL of sterile saline. Post-operatively, mice were monitored twice daily for 24 h to assess general condition (e.g., activity, food/water intake, wound healing, and signs of distress such as hunched posture or lethargy) to ensure timely intervention if abnormal conditions occurred. At 24 h post-CLP, mice were anesthetized with isoflurane prior to cardiac puncture to collect blood samples for subsequent biochemical analysis. Following blood collection, mice were euthanized with an overdose of isoflurane as described above.

### Post-operative monitoring and humane endpoints

Mice were monitored every 6 h for 24 h post-operation (the duration of the experiment) to assess health status and behavioral changes. Monitoring indicators included general activity, food and water intake, body posture, wound healing, respiratory rate, and signs of distress (e.g., hunched posture, lethargy, piloerection, abnormal vocalization, or inability to move freely). Humane endpoints were predefined based on clinical and behavioral criteria: (1) >20% loss of baseline body weight; (2) persistent lethargy or inability to reach food/water; (3) severe abdominal distension or wound infection (redness, swelling, or exudate); (4) respiratory distress (dyspnea or tachypnea); or (5) persistent hypothermia (body temperature <35 °C). If any mouse met these humane endpoint criteria, it was immediately euthanized to minimize suffering. During the 24-h observation period, no mice died before meeting the predefined criteria or reaching the experimental endpoint.

### ELISA

According to the instructions of the ELISA kit, the serum of mice was added into the sample wells of the plate, and the corresponding standard wells were set up simultaneously. The plate was gently shaken and then incubated at 37°C for 2 h. After washing and drying, the biotin-labeled antibody working solution was added. Afterwards, the plate was incubated at 37°C for 1 h, followed by drying and washing three times. Finally, chromogenic substrates A and B were added to each well, and the optical density (OD) value of each well was measured at 450 nm wavelength by a plate reader within 10 minutes after adding the stop solution. The concentrations of Cholinesterase, IL-6, TNF-α, and IL-1β were then calculated.

### Preparation of Splenic Single-Cell Suspension

At 24 hours post-CLP, the mice were sacrificed, and their spleens were harvested. The harvested spleens were immersed in pre-chilled flow cytometry staining buffer. To prepare the splenic single-cell suspension, a frosted glass slide was first moistened with this buffer. The spleens were then mechanically dissociated using the buffer-moistened slide. The resulting cell suspension was collected and transferred into 5 mL centrifuge tubes. After filtration and centrifugation, the supernatant was discarded, and red blood cells (RBCs) were lysed. Subsequently, the cells obtained after RBC lysis were diluted to an appropriate concentration, and cell counting was performed.

### Flow cytometry

Once single-cell suspensions were obtained, cells were stained for flow cytometric analysis. Appropriate volumes of anti-mouse CD4-FITC, CD8-PE, and CD3-APC antibodies were added to each tube of cell suspension. The mixture was incubated at 4°C for 35 minutes in the dark. After washing, cell membrane permeabilization and fixation were performed as two separate steps. Subsequently, an appropriate volume of anti-mouse Foxp3-APC antibody was added, and the samples were incubated again at 4°C for 35 minutes in the dark. Following another washing step, the cells were resuspended in 200–400 μL of FCS solution. Data acquisition was conducted using a BD FACS Canto II flow cytometer.

### Real‑time quantitative PCR (RT‑qPCR)

Total RNA was extracted using TRIzol reagent, and the isolated RNA was reverse transcribed into cDNA using a PrimeScript™ One Step RT-qPCR Kit. RT-qPCR was performed using a PrimeScript™ RT Reagent Kit on an ABI 7900 Real-Time PCR System. The relative gene expression levels were normalized to GAPDH, and the relative quantitative analysis was conducted using the 2^−ΔΔ^CT method. The primer sequences used were as follows: RORA forward 5′-TCTCCCTGCGCTCTCCGCAC-3′, reverse 5′-TCCACAGATCTTGCATGGA-3′; GAPDH forward 5′-AGGTCGGTGTGAACGGATTTG-3′, reverse 5′-GGGGTCGTTGATGGCAACA-3′.

## Statistical Analysis

Normally distributed quantitative data are expressed as mean ± standard deviation, and between-group comparisons were performed using independent samples t-test. Non-normally distributed data are presented as median (interquartile range), and between-group comparisons were conducted using the Mann-Whitney U test. Categorical variables are reported as numbers (percentages), and between-group comparisons were performed using the chi-square test. For clinical subgroup analysis, ChE was dichotomized at a cutoff value of 5000 U/L: low ChE group (< 5000 U/L) and normal ChE group (≥ 5000 U/L). Spearman’s rank correlation analysis was used to evaluate the associations between ChE activity and continuous immune indicators. The diagnostic values of ChE and RORA were assessed using receiver operating characteristic (ROC) curves. All statistical analyses were performed using R software (v3.6.3), and all visualizations were generated using the R package ggplot2. Statistical significance thresholds were uniformly defined as follows: for differential gene expression, P < 0.05 and |log2FC| > 1; for gene set enrichment analysis, |NES| > 1 and FDR q < 0.25; for all other statistical tests, P < 0.05.

## Result

**Fig 1:**
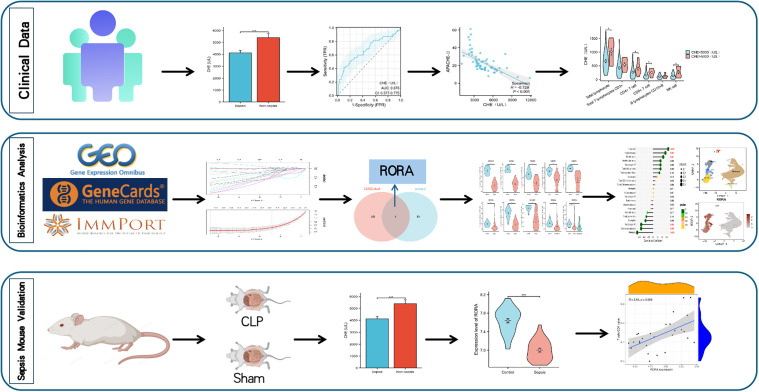
Flowchart of This Study.

The design flowchart for this study is shown in Fig 1.

### Patient characteristics

A total of 148 patients were included in the final analysis, comprising 65 patients with sepsis and 83 control patients without sepsis. As summarized in Table 1, the two groups were well balanced at baseline. There were no significant differences in age (median (IQR): 73.5 (60.0–80.75) vs. 73.0 (56.5–82.5) years, P = 0.535) or sex distribution (P = 0.761). Disease severity, measured by APAChE II score, was also similar between groups (18.0 (15.0–25.0) vs. 17.5 (16.0–23.25), P = 0.857). Additionally, most laboratory parameters—including lactate, CRP, albumin, bilirubin, liver enzymes, and creatinine—showed no significant differences (all P > 0.05). In line with expectations based on prior studies, patients with sepsis exhibited significantly higher levels of procalcitonin (PCT) (3.49 (0.75–75.98) vs. 0.57 (0.08–6.39) ng/mL, P = 0.015) compared to controls.

**Table 1:** Comparison of Baseline Clinical Characteristics Between Sepsis and Non-Sepsis Groups.

Characteristics	Sepsis	Non-sepsis	P value
N	65	83	
Age (years)	73.5 (60, 80.75)	73 (56.5, 82.5)	0.535
Sex, n (%)			0.761
Female	25 (38.4%)	30 (36.1%)	
Male	40 (61.6%)	53 (63.9%)	
Lac (mmol/L)	2.51 (1.25, 5.68)	1.95 (1.30, 3.02)	0.276
PCT(ng/ml)	3.49 (0.75, 75.98)	0.57 (0.08, 6.39)	**0.015**
CRP(mg/L)	69 (63.5, 179)	58.25 (13.825, 148.3)	0.130
APACHE-Ⅱ	17.5 (16, 23.25)	18 (15, 25)	0.857
ALB(g/L)	32.394 ± 5.9352	32.278 ± 5.718	0.914
TBIL(umol/L)	14.65 (9.925, 20.2)	13.35 (9.175, 19.675)	0.572
DBIL (umol/L)	7.8 (4.325, 11.4)	6.35 (3.775, 11.15)	0.288
ALT(U/L)	22 (15.25, 45.25)	19.5 (15, 28.5)	0.353
AST(U/L)	31 (21.25, 56)	25.5 (17.25, 36.75)	0.092
LDH(U/L)	232.5 (181.25, 376)	221.5 (162.25, 276.75)	0.092
ChE (U/L)	4197 (3365, 5268)	4825 (3753.5, 6474)	**0.037**
Cr(umol/L)	106.5 (68.25, 169.5)	99 (61.5, 152.75)	0.549

Lac, lactate; PCT, procalcitonin; CRP, C-reactive protein; APACHE-II, Acute Physiology and Chronic Health Evaluation II; ALB, albumin; TBIL, total bilirubin; DBIL, direct bilirubin; ALT, alanine transaminase; AST, aspartate transaminase; LDH, lactate dehydrogenase; ChE, cholinesterase; Cr, creatinine.

### Serum ChE Levels and Its Diagnostic Performance for Sepsis

**Fig 2:**
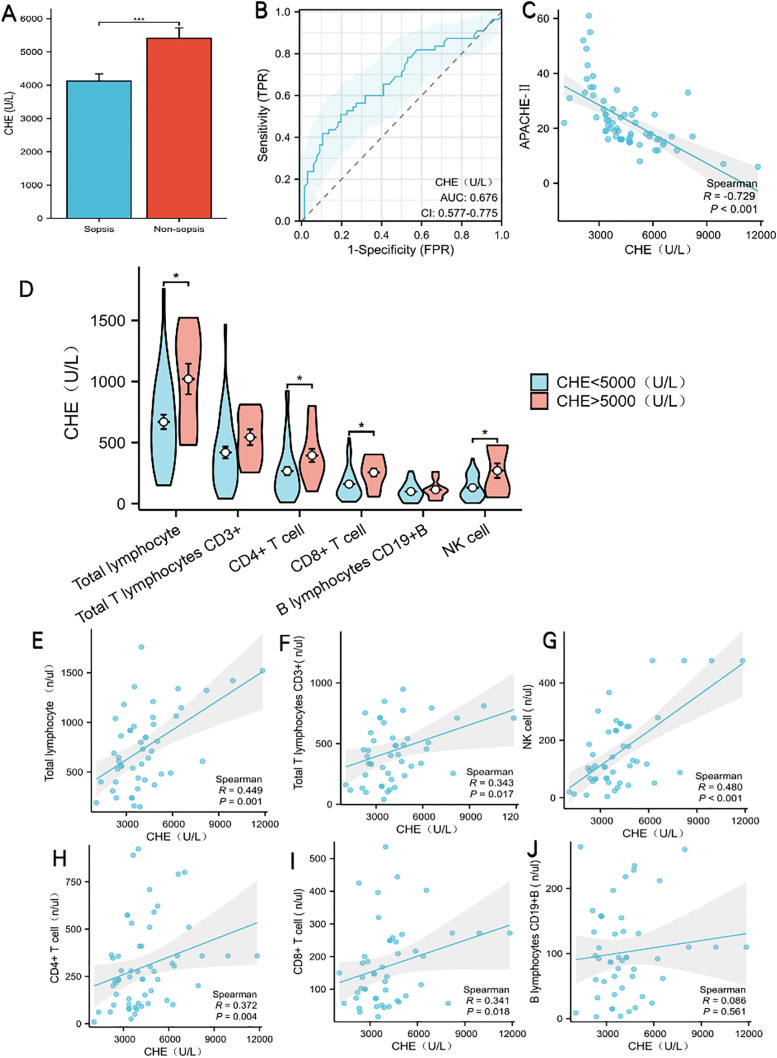
Expression characteristics, diagnostic value, and correlation with immune cells of serum cholinesterase (CHE). (A) Comparison of serum CHE activity between sepsis and non-sepsis groups. (B) ROC curve for CHE in diagnosing sepsis.

Serum ChE activity was significantly lower in the sepsis group than in non-sepsis controls (4197 (3365–5268) vs. 4825 (3753.5–6474) U/L, P = 0.037, Fig 2A). To further evaluate the diagnostic potential of ChE for identifying sepsis, we constructed a receiver operating characteristic (ROC) curve. This analysis yielded an area under the curve (AUC) of 0.676 (95% confidence interval: 0.577–0.775), indicating a moderate discriminative ability of ChE to distinguish sepsis patients from non-sepsis controls (Fig 2B).

### Subgroup analysis based on serum cholinesterase activity

To explore the relationship between ChE levels and clinical features in sepsis patients, 65 sepsis patients were stratified into two subgroups: ChE < 5000 U/L (n = 45) and ChE > 5000 U/L (n = 20). The results showed that no significant differences were observed in gender, lactate (Lac), PCT, CRP, TBIL, DBIL, ALT, AST, LDH, Cr, or 30-day mortality between subgroups (all P > 0.05, Table 2).

**Table 2:** Comparison of Baseline Clinical Characteristics Between CHE < 5000 (U/L) and CHE > 5000 (U/L) in Sepsis Groups.

Characteristics	CHE < 5000 (U/L)	CHE > 5000 (U/L)	P value
N	45	20	
Age (years)	73.22 ± 15.48	64.6 ± 14.536	0.039
Sex, n (%)			0.702
Female	18 (27.7%)	7 (10.8%)	
Male	27 (41.5%)	13 (20%)	
Lac (mmol/L)	2.70 (1.32, 5.62)	2.40 (1.28, 5.10)	0.17
PCT(ng/ml)	3.62 (0.80, 72.10)	2.90 (0.78, 12.50)	0.109
CRP(mg/L)	66.75 (62.85, 185.6)	77.3 (63.5, 90.6)	0.874
APACHE-Ⅱ	23 (18, 32)	16 (12, 18)	**< 0.001**
SOFA score	8 (5–10)	5 (3–9)	**0.027**
ALB(g/L)	30.556 ± 5.097	36.6 ± 5.7984	**< 0.001**
TBIL(umol/L)	12.1 (9.3, 19.4)	15.7 (13.175, 23.25)	0.296
DBIL (umol/L)	9.1 (4.8, 12.5)	5.45 (3.95, 8.325)	0.108
ALT(U/L)	22 (13, 28)	23 (17.75, 83.25)	0.277
AST(U/L)	28 (21, 47)	32 (23.75, 77.75)	0.213
LDH(U/L)	238 (175, 382)	222 (210, 376)	0.532
Cr(umol/L)	116 (80, 193)	86 (66, 108.25)	0.095
30-day mortality	11 (24.4%)	3 (15.0%)	0.672

Lac, lactate; PCT, procalcitonin; CRP, C-reactive protein; APACHE II, Acute Physiology and Chronic Health Evaluation II; SOFA, Sequential Organ Failure Assessment; ALB, albumin; TBIL, total bilirubin; DBIL, direct bilirubin; ALT, alanine transaminase; AST, aspartate transaminase; LDH, lactate dehydrogenase; Cr, creatinine.

However, patients in the ChE < 5000 U/L subgroup were significantly older (73.22 ± 15.48 vs. 64.6 ± 14.54 years, P = 0.039) and had lower serum albumin levels (30.56 ± 5.10 vs. 36.6 ± 5.80 g/L, P < 0.001) (Table 2). They also exhibited greater disease severity, as indicated by higher APAChE-II scores (P < 0.001) and higher SOFA scores (P = 0.027) (Table 2). Furthermore, we investigated the relationship between ChE levels and disease severity in sepsis patients and found a strong negative correlation with APAChE-II scores (Spearman R = −0.729, P < 0.001, Fig 2C). These findings indicate that sepsis patients with reduced ChE levels exhibit a more critical disease status, as reflected by worse disease severity scores and poorer nutritional status.

### Inflammatory and immune indices between ChE subgroups in Sepsis patients

To explore inflammatory and immune profiles in sepsis patients by stratifying them according to ChE levels, we compared relevant indices between subgroups. No significant differences were observed in cytokines, including IL-1β, IL-6, IL-8, IL-10, IFN-γ, TNF-αand IFN-α (all P > 0.05), or in B lymphocyte counts (P = 0.542) and Total T lymphocyte counts (P = 0.091) between the two groups (Table 3). In contrast, the ChE < 5000 U/L subgroup exhibited significantly lower total lymphocyte counts (669.05 ± 364.38 vs. 1020.7 ± 396.09 cells/μL, P = 0.010), CD4 + T cell counts (229 [93.75, 335.75] vs. 342 [244.75, 359] cells/μL, P = 0.021), CD8 + T cell counts (130.5 [66.75, 206.75] vs. 242.5 [135.75, 272] cells/μL, P = 0.048), and NK cell counts (105.5 [59, 218.25] vs. 204.5 [119.25, 478] cells/μL, P = 0.024) compared with the ChE > 5000 U/L subgroup(Table 3,Fig 2D). These findings suggest that lower ChE levels in sepsis patients are associated with reduced immune cell populations, which may reflect immune suppression in this population.

**Table 3:** Inflammatory factor levels and immune cell counts on EICU admission stratified by CHE activity.

Characteristics	CHE < 5000 (U/L)	CHE > 5000 (U/L)	P value
N	45	20	
CHE (U/L)	3502 (2679, 4197)	6248 (5759, 7117.5)	< 0.001
IL-1β (pg/ml)	2.3608 ± 0.6521	2.5233 ± 1.0709	0.678
IL-6 (pg/ml)	101.26 (28, 237.7)	47.89 (30.455, 279.13)	0.774
IL-8 (pg/ml)	35.75 (19.03, 78.11)	32.53 (17.33, 61.08)	0.824
IL-10 (pg/ml)	18.04 (5.8675, 38.76)	53.63 (7.73, 214.54)	0.613
IFN-γ (pg/ml)	4.85 (2.575, 6.86)	4.35 (3.16, 5.04)	0.824
α-TNF (pg/ml)	8.23 (3.775, 29.74)	7.36 (2.76, 15.21)	0.585
α-IFN (pg/ml)	4.585 (2.99, 6.71)	3.26 (2.92, 7.8)	0.716
Total lymphocyte (/ul)	669.05 ± 364.38	1020.7 ± 396.09	**0.010**
Total T lymphocytes CD3+(/ul)	374 (172, 541.25)	524.5 (367.5, 711)	0.091
CD4 + T cell (/ul)	229 (93.75, 335.75)	342 (244.75, 359)	**0.021**
CD8 + T cell (/ul)	130.5 (66.75, 206.75)	242.5 (135.75, 272)	**0.038**
B lymphocytes CD19 + B (/ul)	99.079 ± 69.843	114.5 ± 73.743	0.542
NK cell (/ul)	105.5 (59, 218.25)	204.5 (119.25, 478)	**0.024**

CHE, cholinesterase; IL, interleukin; IFN, interferon; TNF, tumor necrosis factor; CD, cluster of differentiation; NK, natural killer.

### Correlations between ChE and immune cell counts

Correlation analyses (Fig 2E–2J) further clarified the relationship between ChE levels and immune cell subsets. ChE was positively correlated with total lymphocytes (Spearman R = 0.449, P = 0.001), total CD3 + T cells (Spearman R = 0.343, P = 0.017), NK cells (Spearman R = 0.480, P < 0.001), CD4 + T cells (Spearman R = 0.372, P = 0.004), and CD8 + T cells (Spearman R = 0.341, P = 0.018). In contrast, no significant correlation was detected between ChE and B lymphocyte counts (Spearman R = 0.086, P = 0.561). These results collectively highlight that reduced ChE levels in sepsis are closely associated with decreased counts of key cellular immune components. This finding supports the notion that ChE reduction may be linked to immunosuppression in sepsis.

### Identification of candidata hub DEGs and analysis of GO and KEGG pathway enrichment

**Fig 3:**
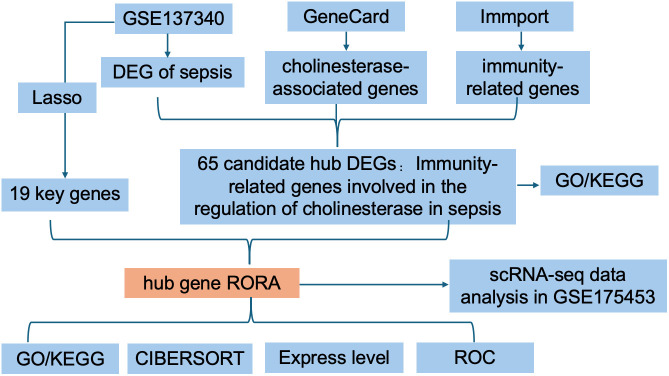
Flowchart of the study design.

To further elucidate the functional role and underlying mechanisms of cholinesterase in sepsis-associated immunity, we performed bioinformatics analyses by integrating sepsis patient datasets, CAPRGs, and IRGs. The diagram of the research design flow is presented in Fig 3.

**Fig 4:**
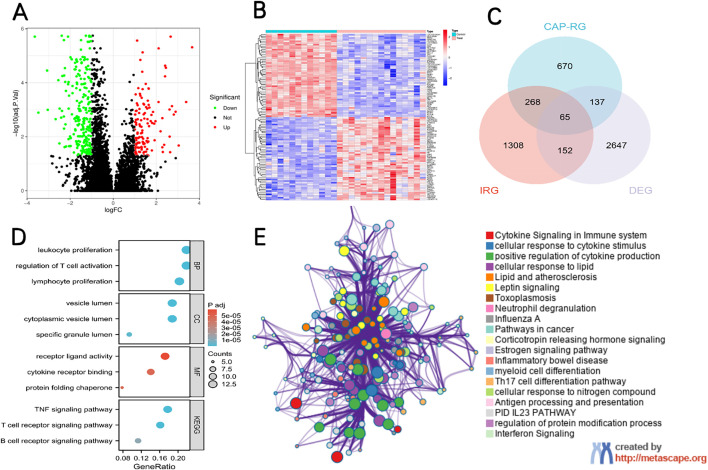
Identification of candidate Hub DEGs and analysis of functional enrichment. **(A)** Volcano plot of DEGs in the GSE137340 dataset. **(B)** Heat map of DEGs in the GSE137340 dataset. **(C)** Venn diagram of candidate hub genes. **(D)** Top 3 enriched GO biological-process (BP) terms of candidate hub genes. **(E)** Protein-Protein Interaction (PPI) network, Molecular Complex Detection (MCODE) module, and functional enrichment analysis of candidate hub genes.

We initially identified 2247 DEGs between septic patients and control individuals in GSE137340. The volcano plot and heat map of DEGs were shown in Fig 4A,4B. We further obtained 65 candidate hub genes by overlapping genes between DEGS, IRGs and CAPRGs (Fig 4C), and they were utilized for GO and KEGG analysis. The top 3 enriched GO terms were listed in Fig 4D. The most significant BP terms were about Leukocyte proliferation, Regulation of T cell activition and Lymphocyte proliferation. We also detected that the hub DEGs were significantly enriched in the Immune and inflammatory mediator pathway by using the Metascape database, the main result of analysis of PPI, MCODE and the function enrichment of hub DEGs were showed in Fig 4E.

### Screening of hub DEG and analysis of functional enrichment

**Fig 5:**
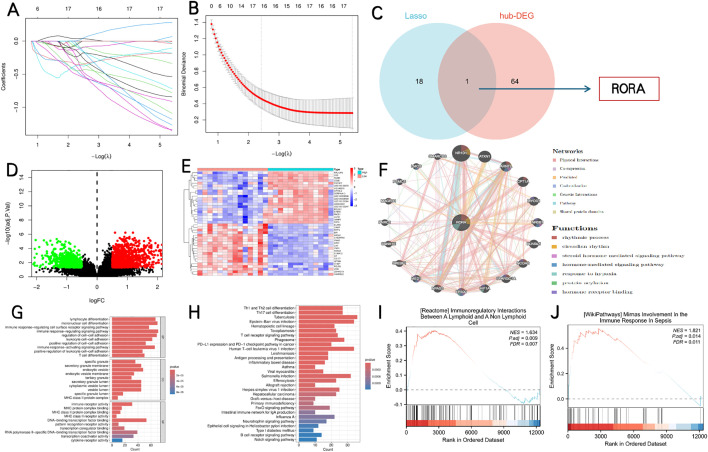
Identification of hub differentially expressed genes (DEGs) and analysis of functional enrichment. **(A, B)** LASSO algorithm-based variable selection showing coefficient plots and partial likelihood deviance. **(C)** The Venn diagram showing overlapping genes leading to the identification of the hub gene RORA. **(D, E)** Volcano plot and heat maps of the DEGs between the high and low RORA expression groups. **(F)** The top 20 genes associated with RORA are shown. **(G, H)** GO and KEGG pathway analyses of the DEGs between the high and low RORA expression groups. **(I, J)** GSEA of the DEGs between the high and low RORA expression groups.

Through LASSO analysis, 19 key genes were identified (Fig 5A,5B). Finally, we obtained the hub DEG RORA by overlapping the candidate hub genes and key genes (Fig 5C).

#### Single-gene analysis for the RORA.

**Identification of DEGs and GSEA analysis.** In the septic sample of GSE137340 dataset, there were 1553 DEGs between RORA high expression group and RORA low expression group. The volcano plot and heat maps of the DEGs were shown in Fig 5D,5E. These DEGs were also analysed by R language for GO and KEGG as in Fig 5G,5H. Top 20 genes associated with RORA were predicted in the GeneMANIA database, such as NR1D1, ATXN1, ARNTL, CTP1A, etc. Their common functions included rhythmic process, circadian rhythm, steroid hormone mediated signaling pathway, hormone-mediated signaling pathway, response to hypoxia, protein acylation, hormone receptor bingding (Fig 5F). Additionally, GSEA mainly identified immunoregulatory Interactions Between A Lymphoid and A Non Lymphoid Cell and Mirnas Involvement In the Immune Response In Sepsis (Fig 5I,5J).

**Fig 6:**
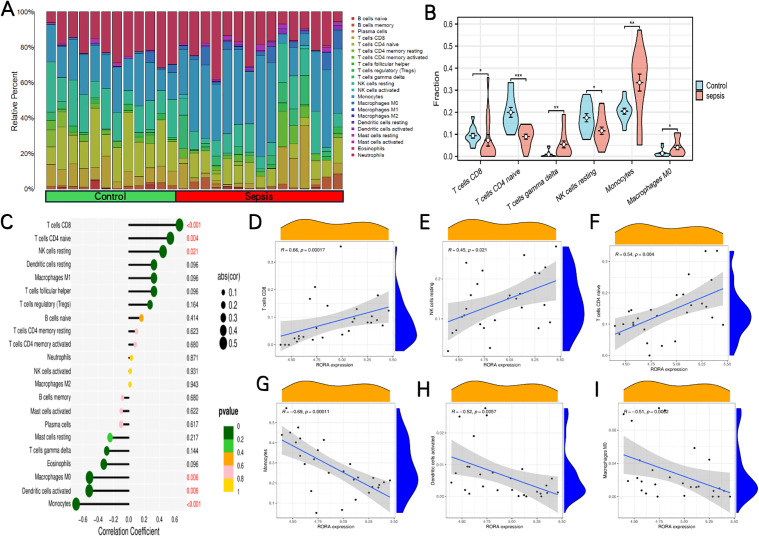
Immune infiltration analysis and correlation with RORA. **(A)** CIBERSORT analysis of 22 immune cell types in control and sepsis groups. **(B)** Differential immune cell infiltration between control (blue) and sepsis (pink) groups. **(C)** Correlation analyses between RORA and immune cell types. Point size represents correlation coefficient magnitude; colour indicates statistical significance. (D–I) Correlation analyses between RORA and CD8 + T cells, resting NK cells, naive CD4 + T cells, monocytes, activated dendritic cells, and M0 macrophages. (*P < 0.05; **P < 0.01; ***P < 0.001).

**Correlation between RORA and immune cell infiltrates.** Based on immune cell infiltration analysis, we used the CIBERSORT algorithm to evaluate the composition of 22 immune cell types in the control and sepsis groups. A cumulative bar chart illustrated the relative proportion of each immune cell subtype (Fig 6A), revealing significant alterations in the proportions of T cells CD8, T cells CD4 naïve, T cells gamma delta, NK cells resting, Monocytes, Macrophages M0 (P < 0.05) (Fig 6B). Differential analysis showed that the proportions of T cells gamma delta, Monocytes and Macrophages M0 were significantly increased in sepsis group (P < 0.05), whereas the proportions of T cells CD8, T cells CD4 naïve and NK cells resting were significantly decreased (P < 0.05). These findings provide important insights into the immune microenvironment characteristics of sepsis.

RORA showed a significant positive correlation with T cells CD8, T cells CD4 naïve, NK cells resting (P < 0.05) (Fig 6C). To validate these associations, we performed regression analyses between RORA expression and key immune cells (Fig 6D-6I). RORA expression was positively correlated with CD8 + T cells (R = 0.66, P = 0.00017), NK resting cells (R = 0.45, P = 0.021), and naive CD4 + T cells (R = 0.54, P = 0.004). Conversely, RORA was negatively correlated with monocytes (R = −0.69, P = 0.00011), activated dendritic cells (R = −0.52, P = 0.0057), and M0 macrophages (R = −0.51, P = 0.0062). Taken together, these results indicate that RORA expression is closely linked to the composition of adaptive and innate immune cell populations, suggesting that RORA may play a regulatory role in modulating immune cell composition within the tissue microenvironment.

#### Validation of RORA expression and diagnostic value.

**Fig 7:**
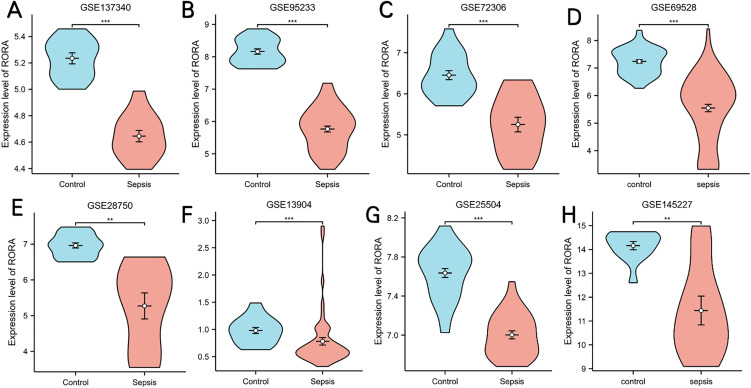
Comparison of RORA expression levels between the sepsis group and the control group. **(A)** Comparison of RORA expression levels between the sepsis group and the control group within the GSE137340 dataset. **(B)** Comparison of RORA expression levels between the sepsis group and the control group within the GSE95233 dataset. **(C)** Comparison of RORA expression levels between the sepsis group and the control group within the GSE72326 dataset. **(D)** Comparison of RORA expression levels between the sepsis group and the control group within the GSE69528 dataset. **(E)** Comparison of RORA expression levels between the sepsis group and the control group within the GSE28750 dataset. **(F)** Comparison of RORA expression levels between the sepsis group and the control group within the GSE13904 dataset. **(G)** Comparison of RORA expression levels between the sepsis group and the control group within the GSE25504 dataset. **(H)** Comparison of RORA expression levels between the sepsis group and the control group within the GSE145227 dataset.

**Fig 8:**
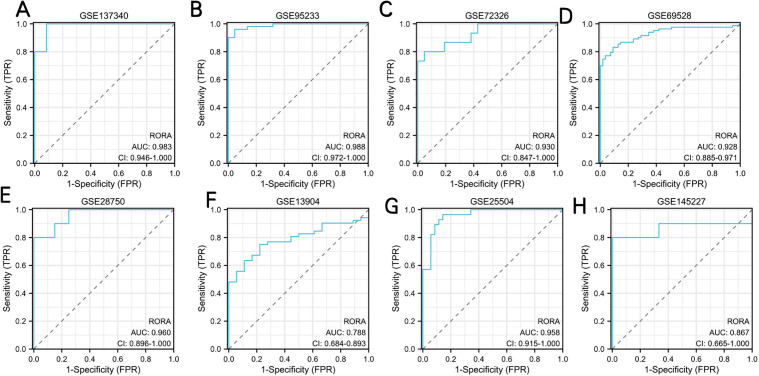
Diagnostic potential of RORA in sepsis. **(A) The diagnostic potential of RORA in sepsis within the GSE137340 dataset. (B)**The diagnostic potential of RORA in sepsis within the GSE95233 dataset. **(C)**The diagnostic potential of RORA in sepsis within the GSE72326 dataset. **(D)**The diagnostic potential of RORA in sepsis within the GSE69528 dataset. **(E)**The diagnostic potential of RORA in sepsis within the GSE28750 dataset. **(F)**The diagnostic potential of RORA in sepsis within the GSE13904 dataset. **(G)**The diagnostic potential of RORA in sepsis within the GSE25504 dataset. **(H)**The diagnostic potential of RORA in sepsis within the GSE145227 dataset.

In order to verify the reliability of RORA expression levels, we added another 7 datasets containing sepsis and control samples and analyzed RORA expression levels. The outcome suggested that RORA expression was significantly downregulated in sepsis samples compared with control samples in all 8 datasets (Fig 7). To assess the diagnostic utility of RORA for sepsis, we analyzed its ROC curves across 8 independent GEO datasets (Fig 8). The result showed that RORA had high diagnostic value for sepsis. The AUC of RORA was 0.983 (95% CI: 0.946–1.000) in GSE137340, 0.998 (95% CI: 0.972–1.000) in GSE95233, 0.930 (95% CI: 0.847–1.000) in GSE72326, 0.928 (95% CI: 0.885–0.971) in GSE69528, 0.960 (95% CI: 0.896–1.000) in GSE28750, 0.788 (95% CI: 0.684–0.893) in GSE13904, 0.958 (95% CI: 0.915–1.000) in GSE25504, and 0.867 (95% CI: 0.665–1.000) in GSE145227. The robust diagnostic performance of RORA further supports its reliability as a biomarker for sepsis and immunosuppression, and enables combined evaluation with serum ChE for clinical application.

### Six cell types were annotated by scRNA-seq data analysis

**Fig 9:**
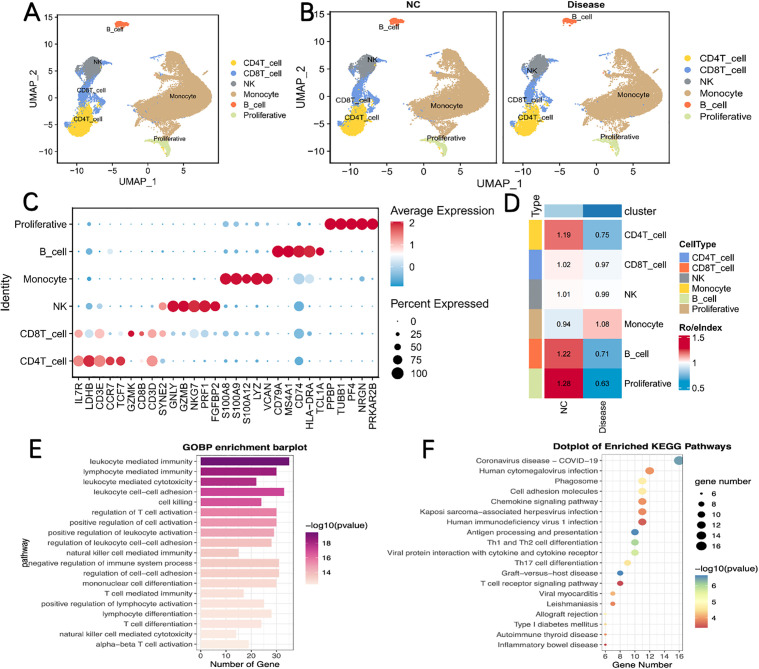
Six cell types were annotated through scRNA-seq data analysis. **(A)** UMAP dimensionality reduction clustering results and cell subpopulation annotations; **(B)** Distribution of cell subpopulations across two groups; **(C)** Bubble plot showing marker gene expression across the six cell types; **(D)** Cell proportion plots within different groups. **(E, F)** Enriched GO and KEGG pathways in each cell type.

To further clarify the cellular localization and expression patterns of RORA in specific immune cell subsets (CD4 ⁺ T, CD8 ⁺ T, NK cells) that are core to our ChE‑mediated immunosuppression hypothesis, we analyzed the single-cell RNA sequencing dataset GSE175453, which included peripheral blood mononuclear cells from 5 healthy controls and 4 septic patients. We then performed reduced-dimensional clustering analysis and identified 6 main cell types: CD4 ⁺ T cells, NK cells, CD8 ⁺ T cells, B cells, monocytes, and proliferative cells (Fig 9A). These cell types were distributed in both the NC group and sepsis group (Fig 9B). The expression of marker genes for these cell types is shown in the bubble plot (Fig 9C). We also observed significant differences in the proportion of CD4 ⁺ T cells, NK cells, CD8 ⁺ T cells, B cells, monocytes, and proliferative cells between the two groups (Fig 9D). Furthermore, GO and KEGG enrichment analyses were performed using the clusterProfiler package, and the results showed significant differences in enriched pathways among different cell subsets (Fig 9E, 9F).

### Cell communication analysis

**Fig 10:**
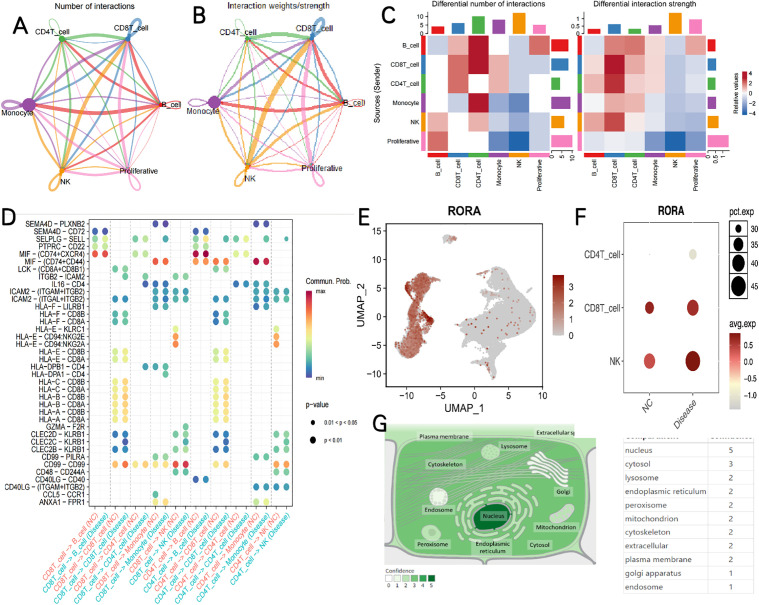
Communication between key cells and other cells and expression of key genes in different cells. **(A,B)** Chord diagram depicting differences in the number and intensity of cell-cell communication interactions among sepsis cell subsets. **(C)** Heatmap depicting differences in the number and intensity of cell-cell communication interactions among sepsis cell subsets. **(D)** Bubble chart of cell communication. **(E)** Expression of RORA in different cells. **(F)** The expression levels of RORA was significantly different in two groups. **(G)** RORA subcellular localization.

Cell communication analysis was implemented to probe the exchange of information among the six cell types identified by annotation. There was an increased number and intensity of interactions among CD4 + T cells, NK cells, CD8 + T cells, and B cells in the sepsis groups (Fig 10A-10C). Furthermore, our research findings indicate that when comparing the NC group with the disease group, there are significant differences in the cell communication between CD8 + T cells and NK cells, as well as between CD4 + T cells and NK cells, both mediated by the HLA-E-CD94 and HLA-E-KLRC1 pathways (Fig 10D). The expression levels of RORA were evaluated in cells identified by annotation. The results demonstrated that RORA was highly expressed in NK cells, CD4 + T cells, and CD8 + T cells (Fig 10E). Subsequently, the expression of RORA was compared in these cell types between the NC and disease groups. The results indicated that the expression levels of RORA in the disease group were significantly higher in NK cells, CD4 + T cells, and CD8 + T cells than in the NC group (Fig 10F). To determine the specific locations of RORA in cells, we predicted the sub-cellular localization of RORA using the COMPARTMENTS data base. The results showed that RORA was mainly distributed in the nucleus and cytosol (Fig 10G).

Of note, the elevated RORA expression observed within specific immune cell subsets (NK cells, CD4 ⁺ T cells, CD8 ⁺ T cells) in the single-cell analysis does not conflict with the overall downregulation of RORA in bulk transcriptome and animal models. The single-cell data reflect the relative expression level of RORA within the positive cell subsets, whereas the bulk sequencing data represent the overall average expression level in the entire tissue or blood. In sepsis, the absolute counts of CD4 ⁺ T, CD8 ⁺ T, and NK cells decrease significantly, which leads to an overall reduction in total RORA expression at the bulk level. These two findings reflect different analytical dimensions and are mutually supportive rather than contradictory.

### Flow cytometry analysis of immunological cell changes

**Fig 11:**
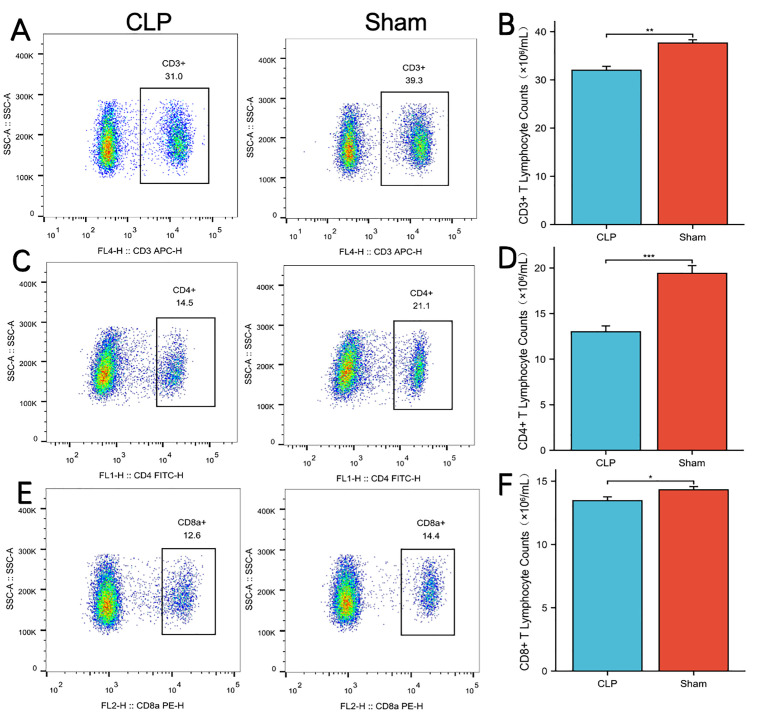
Flow cytometry measured the absolute counts of CD4 + T cells, CD8 + T cells, and CD3 + T cells. (A, C, **E)** Representative flow cytometry contour plots of CD3+ **(A)**, CD4+ **(C)**, and CD8+ **(E)** T cells in the spleen from CLP mice (left) and Sham mice (right). (B, D, **F)** Quantification of absolute counts (×10³/mL) for spleen CD3+ **(B)**, CD4+ **(D)**, and CD8+ **(F)** T cells in CLP and Sham groups. Statistical significance: *P < 0.05, **P < 0.01, ***P < 0.001.

To characterize immune cell changes in sepsis, we measured the counts of CD3 ⁺ T cells, CD4 ⁺ T cells, and CD8 ⁺ T cells in the spleens of CLP and Sham mice using flow cytometry. Results showed that compared with Sham mice, the counts of CD3 ⁺ T cells, CD4 ⁺ T cells, and CD8 ⁺ T cells in the spleens of CLP mice were significantly reduced (Fig 11) (all P < 0.05).

### Validation of ChE concentration and RORA mRNA expression

**Fig 12:**
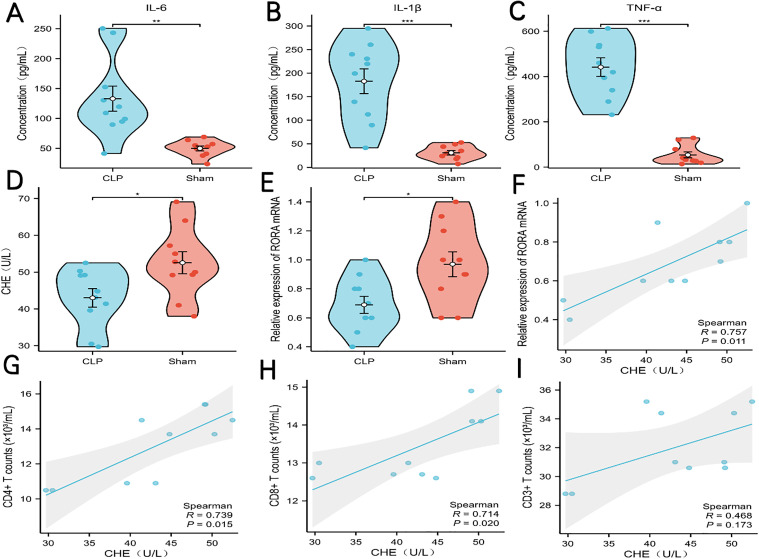
Validation of CHE Concentration and RORA mRNA Expression in CLP Mice. **(A–C)** Violin plots showing concentrations of serum IL-6 **(A)**, IL-1β **(B)**, and TNF-α (C) in CLP and Sham groups; **(D)** Violin plot of serum cholinesterase (CHE) activity in CLP and Sham groups; **(E)** Bar graph depicting relative mRNA expression of RORA in the spleen of Sham and CLP groups; **(F)** Correlation plot showing the relationship between RORA mRNA expression and CHE Concentration. (G-I) Correlation plot showing the relationship between CD4 ⁺ T, CD8 ⁺ T, CD3 ⁺ T cell counts and CHE concentration. Statistical significance: *P < 0.05, **P < 0.01, ***P < 0.001.

To further validate alterations in ChE concentration and RORA mRNA expression levels, we detected ChE concentration in serum using ELISA and assessed RORA mRNA expression levels in spleen tissue via PCR in both CLP and Sham mice. IL-6, IL-1β, and IFN-γ concentrations were significantly elevated in CLP mice compared to Sham mice (P < 0.05, Fig 12A-12C). ChE concentration was significantly reduced in the CLP group compared to the Sham group (P < 0.05, Fig 12D), and RORA mRNA expression level was also significantly reduced in the CLP group compared to the Sham group (P < 0.05, Fig 12E). Reduced ChE expression in CLP mice correlates with our clinical findings, and the expression results of RORA was also consistent with the trend observed in microarray analysis. Additionally, correlation analysis indicated a positive correlation between ChE concentration and RORA mRNA expression levels(Fig 12F).

### Correlation analysis of ChE concentration with immune cells in CLP mice

To further investigate the correlation between ChE concentration and mainly T cell counts in CLP mice, we analyzed their relationship. The results showed that in CLP mice, serum ChE concentration correlated positively and significantly with both CD4 ⁺ T cells (Spearman R = 0.739, P = 0.015) and CD8 ⁺ T cell counts (Spearman R = 0.714, P = 0.020), but showed no significant correlation with CD3 ⁺ T cell counts (Spearman R = 0.468, P = 0.173) (Fig 12G-12I).

## Discussion

The essence of sepsis lies in the host’s dysregulated immune response to infection [17]. This profound immune dysfunction not only leads to multiple organ dysfunction syndrome (MODS), but also significantly increases the risk of patient mortality [18,19]. Sepsis-induced immunosuppression represents a major cause of death in affected patients. However, effective biomarkers for the early identification and intervention of sepsis-induced immunosuppression remain lacking. Therefore, a deeper understanding of the immune and inflammatory mechanisms underlying sepsis and identifying relevant biomarkers are crucial to improve diagnostic accuracy, predict disease progression, and guide personalized treatment strategies.

In our clinical practice in the intensive care unit, we observed that serum cholinesterase (ChE) levels were widely decreased in patients with sepsis. To clarify these relationships and verify our hypothesis that the reduction in ChE may be associated with sepsis-induced immunosuppression, we performed a comprehensive study integrating clinical cohort analysis, bioinformatics analysis, and animal experiments. Clinically, we confirmed that serum ChE activity was significantly reduced in sepsis patients. The decreased ChE level was significantly correlated with higher disease severity (APACHE II and SOFA scores) and reduced counts of key immune cells, including CD4 ⁺ T cells, CD8 ⁺ T cells, and NK cells. ChE also showed moderate diagnostic value for sepsis. These findings suggest that ChE may serve as a potential biomarker for sepsis and may be involved in sepsis-associated immunosuppression. To further explore the molecular basis underlying these clinical observations, we performed bioinformatics analyses and identified RORA as a hub immune-related gene involved in ChE-associated pathways during sepsis using multi-dataset validation. RORA expression was downregulated in sepsis patients, and its expression level was positively correlated with the immune infiltration of CD8 ⁺ T cells, CD4 ⁺ T cells, and NK cells. ROC curve analysis confirmed that RORA exhibited high diagnostic value for sepsis, which was consistent with our clinical findings. In the mouse sepsis model, serum ChE concentration and splenic RORA mRNA expression were both significantly reduced in the CLP group compared with the sham group. Both indicators were positively correlated with splenic CD4 ⁺ T and CD8 ⁺ T cell counts.

ChE is widely involved in neural transmission and also plays a critical role in infectious diseases and inflammatory regulation. Previous studies have shown that serum ChE activity is significantly decreased in patients with severe infection or sepsis, and low ChE levels are associated with increased mortality [20,21]. In addition, reduced acetylcholinesterase (AChE) and butyrylcholinesterase (BChE) activities have been reported in both viral and non-viral sepsis, with predictive value for the occurrence of sepsis-associated encephalopathy (SAE) and mortality risk [22]. In critically ill patients with COVID-19, decreased serum ChE activity was significantly associated with disease aggravation and increased mortality [20]. Patients with viral sepsis complicated by SAE showed significantly lower AChE activity at 3 days after onset than those without encephalopathy, while BChE activity was negatively correlated with mortality at 7 days [23]. These findings are consistent with our results. Studies have also demonstrated that low ChE activity is significantly correlated with elevated inflammatory markers such as CRP and PCT, suggesting that decreased ChE activity reflects intensified inflammatory responses [20,24]. In summary, ChE acts not only as a neurotransmitter-degrading enzyme but also as a key mediator in inflammatory responses and immune regulation.

It should be noted that serum ChE is mainly synthesized by the liver, and its level can be affected by age, hepatic synthetic function, and nutritional status. In this study, the low ChE group was significantly older and had lower serum albumin levels than the high ChE group. Advanced age and poor nutritional status are known to be associated with impaired immune function and poor prognosis in sepsis. Therefore, we acknowledge that age and albumin may be confounding factors that partially affect the observed association between ChE levels and immune cell counts.

However, after adjusting for the potential effects of age and albumin, serum ChE levels still remained significantly correlated with CD4 ⁺ T cells, CD8 ⁺ T cells, and NK cell counts in our cohort. This indicates that the association of ChE with sepsis-induced immune cell reduction is independent of age and nutritional status. Therefore, serum ChE may serve as a reliable and practical biomarker for assessing sepsis severity and immunosuppression, even after accounting for these important clinical covariates.

The mechanism by which ChE participates in sepsis and inflammatory responses remains incompletely understood. Some scholars have proposed that decreased ChE activity leads to abnormal acetylcholine levels, which affect the vagus nerve–immune axis and disrupt immune homeostasis [25]. By regulating acetylcholine metabolism, ChE modulates acetylcholine-mediated anti-inflammatory pathways. During sepsis, dysregulation of ChE may further exacerbate inflammatory responses. In the progression of sepsis, decreased ChE activity disrupts acetylcholine metabolism, impairs CAP function, and leads to immune disorders, making it difficult to effectively control inflammation [25,26]. Thus, changes in ChE activity not only serve as a biomarker of the inflammatory status in sepsis but may also participate in regulating its pathological process. In addition, ChE activity is closely related to immune cell function, including neutrophil activation and oxygen radical production [26]. Our study revealed that decreased ChE activity was significantly associated with reduced counts of CD4 ⁺ T cells, CD8 ⁺ T cells, and NK cells. Therefore, the reduction in ChE activity may contribute to the immunosuppressive state observed in sepsis.

To further investigate immune-related genes involved in ChE metabolism during sepsis, we identified a hub gene, RORA, through bioinformatics screening and validated it in a CLP mouse model. We confirmed that RORA mRNA expression levels were significantly reduced in CLP mice and positively correlated with the number of CD4 ⁺ T and CD8 ⁺ T cells in the spleen, suggesting that RORA may play an important role in the regulation of immune cells in sepsis.

Retinoic acid-related orphan receptor alpha (RORA), a member of the nuclear receptor superfamily, is a crucial transcription factor. RORA not only participates in modulating the circadian rhythms of organisms but also regulates inflammatory responses and metabolic processes. By suppressing inflammation and mitigating inflammatory damage, RORA plays a fundamental role in tumor development, diabetes, atherosclerosis, rheumatoid arthritis, and other chronic diseases [27–29]. Studies have shown that RORA suppresses the expression of Wnt/β-catenin target genes, thereby attenuating the Wnt/β-catenin signaling pathway, which helps prevent colorectal cancer [30]. Moreover, RORA may also participate in the pathogenesis of breast cancer by inhibiting Wnt/β-catenin gene transcription [31].

Additionally, RORA is widely expressed in various immune cells, participating in their development and playing a crucial role in regulating the body’s immune function [32,33]. Notably, RORA also plays a significant role in the immune regulation and pathogenesis of sepsis. It modulates inflammatory responses and cell differentiation in CD4 ⁺ T cells, and its deficiency leads to impaired immune cell function, affecting sepsis-related immune regulation [33]. In sepsis-induced acute lung injury and acute respiratory distress syndrome (ARDS), RORA exerts important protective effects. It suppresses excessive activation of alveolar macrophages, thereby reducing pulmonary inflammation and edema. Under sepsis conditions, downregulated RORA expression in lung tissue triggers massive production of proinflammatory cytokines by alveolar macrophages, exacerbating pulmonary inflammation [34]. Enhancing RORA activity suppresses macrophage release of inflammatory mediators, mitigates oxidative stress, and promotes lung tissue repair. RORA also regulates circadian rhythm gene expression in lung tissue, maintaining pulmonary immune homeostasis and reducing inflammatory damage. Furthermore, RORA activation promotes repair and regeneration of pulmonary cells, inhibits inflammatory cell infiltration, and mitigates pathological changes in ARDS, thereby improving respiratory function [34]. In studies of sepsis-associated kidney injury, RORA overexpression significantly reduces apoptosis and inflammatory responses by directly binding to the PGC-1α promoter to enhance its transcription, thereby protecting renal tubular cell function [35]. This regulatory mechanism indicates RORA’s involvement in modulating renal cell metabolism and anti-inflammatory responses to mitigate sepsis-associated kidney injury. In the immunopathological mechanisms of sepsis, RORA expression is typically downregulated and significantly correlates with immune cell activity and inflammatory mediator levels, suggesting RORA may play a key regulatory role in sepsis-induced immune dysregulation [36]. Based on these findings, RORA represents not only a crucial molecular target in the pathogenesis of sepsis but also a potential therapeutic target for modulating immune responses, mitigating inflammatory storms, and alleviating multi-organ injury in sepsis treatment.

Of note, our finding that RORA is downregulated in sepsis is consistent with its reported anti‑inflammatory function. Under physiological conditions, RORA provides anti-inflammatory protection by inhibiting inflammatory cell infiltration and proinflammatory cytokine production. In sepsis, the significant downregulation of RORA weakens this protective effect, leading to unrestrained inflammatory responses and elevated inflammatory cytokines, which is consistent with the inflammatory status observed in our model. This further supports that reduced RORA expression contributes to sepsis‑associated inflammation and immunosuppression.

Although the precise mechanistic link between ChE and RORA in immune cells remains to be fully elucidated, we propose a potential biological hypothesis based on existing evidence and our findings. ChE is a key enzyme in the cholinergic anti‑inflammatory pathway that regulates acetylcholine levels. In sepsis, the reduction in ChE activity may disrupt cholinergic signaling and induce an imbalance in inflammatory and immune homeostasis. We hypothesize that ChE deficiency‐induced cholinergic dysregulation may suppress RORA transcription in CD4 ⁺ T cells, CD8 ⁺ T cells, and NK cells. As a critical nuclear receptor and transcription factor, decreased RORA expression further impairs the differentiation, survival, and function of these immune cells, ultimately contributing to sepsis‐induced immunosuppression. This hypothesis provides a plausible explanation for our clinical, bioinformatic, and animal observations. Further experimental studies are warranted to verify this regulatory cascade.

There are several limitations in this study. First, this study employed a single-center retrospective design with a relatively limited sample size, potentially introducing selection bias, and the clinical data did not include long-term prognostic information for patients, preventing an assessment of the predictive value of ChE and RORA for long-term sepsis outcomes. Second, the animal experiments only measured ChE activity and RORA expression at a single time point (24 hours after CLP), which precluded analysis of the temporal dynamic relationship between ChE and RORA. Third, the present study demonstrated a significant correlation between ChE and RORA but did not provide direct mechanistic evidence (e.g., gain‑ or loss‑of‑function assays) confirming that ChE regulates RORA expression. Therefore, our conclusion that RORA may serve as a potential molecular bridge linking ChE to sepsis‑associated immunosuppression is supported by correlational evidence rather than direct causal proof. Further mechanistic studies with time‑series sampling and functional validation are warranted to verify this regulatory cascade.

## Conclusion

Combining clinical cohort analysis, bioinformatics analysis, and validation in CLP mice models, this study identifies serum ChE as a biomarker for sepsis diagnosis, severity, and immunosuppression, which is linked to reduced CD4⁺ and CD8 ⁺ T cell counts and NK cell counts. Furthermore, bioinformatics and animal experiments suggest that RORA, a core hub gene downregulated in sepsis, may serve as a molecular bridge connecting ChE activity to T cell dysfunction, and RORA expression positively correlates with both ChE levels and CD4⁺ and CD8 ⁺ T cell counts in septic mice. This work clarifies the mechanisms underlying immune dysregulation in sepsis and supports RORA-targeted immunotherapies as well as optimized clinical immune monitoring.
